# HIF1α Regulates IL17 Signaling Pathway Influencing Sensitivity of Taxane-Based Chemotherapy for Breast Cancer

**DOI:** 10.3389/fcell.2021.729965

**Published:** 2021-09-14

**Authors:** Huijuan Dai, Xiaonan Sheng, Yaohui Wang, Liheng Zhou, Yanping Lin, Yueyao Du, Fan Yang, Rui Sha, Jing Peng, Linli Yao, Wenjin Yin, Jinsong Lu

**Affiliations:** ^1^Department of Breast Surgery, Renji Hospital, School of Medicine, Shanghai Jiao Tong University, Shanghai, China; ^2^State Key Laboratory of Oncogenes and Related Genes, Shanghai Cancer Institute, Renji Hospital, School of Medicine, Shanghai Jiao Tong University, Shanghai, China

**Keywords:** hypoxia, hypoxia-inducible factor 1α, breast cancer, chemotherapy, IL-17 pathway

## Abstract

Hypoxia-induced chemotherapy resistance is the main hindrance for solid tumor treatment. Hypoxia inducible factor-1α (HIF1α), an adaptive gene of hypoxia condition, played an important role in affecting chemotherapy sensitivity for many cancer types and various therapeutic regimens. This study focused on the impact of HIF1α on predicting response and survival of taxane-based neoadjuvant therapy (NAT) for breast cancer (BC) patients and the concrete mechanism that HIF1α mediated paclitaxel chemo-insensitivity. We evaluated HIF1α expression immunohistochemically from biopsies of 108 BC patients receiving paclitaxel–cisplatin NAT. Univariate and multivariate logistic regression analysis revealed that high HIF1α expression led to lower rate of pathological complete response (pCR) and worse prognosis. Analysis of GEO datasets also indicated negative association between HIF1α expression and response of taxane-based NAT in BC patients. The Kyoto Encyclopedia of Genes and Genomes (KEGG) pathway enrichment of differential expression genes (DEGs) in different HIF1α expression groups from TCGA database showed that HIF1α participated in interleukin 17 (IL-17) signaling pathway. Correlation analysis suggested that HIF1α was positively related to the IL-17 pathway. CXC motif chemokine ligand 10 (CXCL10) was the only DEG in the IL-17 pathway inversely relating to NAT response. Experiments *in vitro* verified that HIF1α/IL-17 pathway influences paclitaxel sensitivity to BC cells. Correlation analysis between HIF1α/IL-17A/CXCL10 and infiltration of immune cells in BC uncovered that high expression of all the above three genes were positively correlated to neutrophil infiltration in BC. Collectively, our findings shed novel insight into the mechanism of chemotherapy resistance and implied that HIF1α inhibitor may be a promising drug combined with traditional chemotherapeutic drug to increase the chemotherapy efficacy.

## Introduction

Breast cancer (BC) is the most common malignancy and the leading cause of cancer death in women. Neoadjuvant therapy (NAT) is widely used in locally advanced BC patients as well as patients with early-stage BC for the sake of breast-conserving surgery (BCS) (Guarneri et al., 2006). Pathological complete response (pCR) after NAT predicts the clinical outcomes in BC and can be recognized as the candidate biomarkers for treatment efficacy (Fisher et al., 1998; Kuerer et al., 1999). Taxane-based chemotherapy has been a valuable therapeutic option for both metastatic breast cancer (MBC) (King et al., 2009) and early-stage BC (Bedard et al., 2010) with improved progression-free survival and overall survival (OS) of patients (Caparica et al., 2019). Taxane is also a regular neoadjuvant chemotherapy regimen showing high clinical response rate and pCR rate (Nowak et al., 2004). Nevertheless, inherent and acquired drug insensitivity are the impediment for the better application of taxane for BC patients. As a result, exploring the potential drug resistance mechanisms and blocking them to improve the pCR rate of taxane-based NAT in BC is an imperative mission.

Tumor microenvironment (TME) consists of tumor cells and non-tumor cells such as immune cells and infiltrating inflammatory cells that contribute to tumor development, progression, and response to therapy (Sathe et al., 2020). Hypoxia, destroying microcirculation in structure and function, can significantly alter TME to drive cancer progression by regulating many different pathways (Harris, 2002; Balamurugan, 2016). Hypoxia inducible factor-1α (HIF1α) is an important transcription factor regulating cell adaptation to hypoxia (Rankin and Giaccia, 2016). It has been reported that HIF1α activation in BC patients is a major risk factor for late stage, metastasis, short survival, and poor treatment response (Generali et al., 2006). In terms of BC treatment response, HIF1α expression is associated with endocrine therapy resistance in BC (Jogi et al., 2019; Morotti et al., 2019) and also lapatinib resistance in HER2-positive BC (Karakashev and Reginato, 2015). Furthermore, the stability of HIF1α was closely related to the chemoresistance for triple negative breast cancer (TNBC) patients (Xiong et al., 2018). Overexpression of HIF1α is tightly linked to the insensitivity of MCF-7 cells to doxorubicin treatment (Cao et al., 2013). However, the investigation about the correlation between HIF1α expression and taxane-based NAT efficacy and the role of HIF1α in paclitaxel response for BC is still insufficient.

Tumor microenvironment often occurs in inflammation that can promote cancer cell growth, motility, and metastasis (Mantovani et al., 2008). Pro-inflammatory interleukin 17 (IL-17) has attracted attention for its contribution to reshaping TME (Gorczynski, 2020). It is a cytokine family composed of six interleukins (IL-17A to F) with the ability to promote inflammation (Gaffen, 2009). IL-17A is the hallmark member of IL-17 family relating to BC growth and metastasis (Du et al., 2012). The γδ T cell producing IL-17A can increase neutrophil accumulation in BC TME to promote BC metastasis (Coffelt et al., 2015). Whether IL-17A actively take part in chemotherapy sensitivity is an interesting topic worth investigating.

In this study, we revealed that HIF1α expression was a negative predictor of pCR and survival for BC patients after receiving paclitaxel-based NAT in a registered clinical trial. Then we performed Kyoto Encyclopedia of Genes and Genomes (KEGG) analysis of different HIF1α expression group based on the TCGA database and found that HIF1α was involved in IL-17 pathway. Further correlation between HIF1α expression and differential expression genes (DEGs) participating in IL17 pathway was analyzed. GEO data analysis also confirmed that HIF1α expression was tightly correlated to pCR of taxane-based chemotherapy for BC patients, and IL-17-regulated gene CXC motif chemokine ligand 10 (CXCL10) was the only factor negatively relating to pCR after NAT. *In vitro* experiments demonstrated that HIF1α/IL-17 pathway indeed partially influenced paclitaxel chemosensitivity. Finally, the correlation of HIF1α/IL-17A/CXCL10 and immune cell infiltration in BC was analyzed, and neutrophil infiltration was found to be positively correlated with all three gene overexpression.

## Materials and Methods

### Patient Cohort

Breast cancer patients from two registered clinical trials SHPD001 (NCT02199418) and SHPD002 (NCT02221999) were enrolled in this study. All procedures conducted in this study referring to the human participants were in line with the ethical standards of the institutional and/or national research committee and with the 1964 Helsinki Declaration and its later amendments or comparable ethical standards.

Patients were all qualified with the following criteria: 18 to 70 years old, with large operable BC (pathologically confirmed with biopsy), and without previous systemic therapy for BC or tolerating with chemotherapy. Neoadjuvant chemotherapy regimens were as follows: paclitaxel was administered weekly on day 1 for 16 weeks with a dose of 80 mg/m^2^, and cisplatin was given weekly on days 1, 8, and 15 with a dose of 25 mg/m^2^. Concurrent trastuzumab was administered to the patients if they were HER2 positive. For ER or PR-positive patients, patients in SHPD001 received chemotherapy, while for patients in SHPD002, aromatase inhibitor with or without gonadotropin-releasing hormone agonist was randomized according to their menstrual status. Planned surgery was performed sequentially after NAC. The primary outcome of SHPD001 and SHPD002 was pCR, which was defined as the absence of invasive tumors in the breast and axillary lymph node samples after NAT. Detailed information of the clinical trial is also described in a previous article and ClinicalTrials.gov.

### Immunohistochemical Staining

Immunohistochemistry of HIF1α was conducted according to a previous study (Jiang et al., 2019). Mouse monoclonal HIF1α primary antibody was employed (Abcam, United States). HIF1α expression was evaluated according to the following criteria: += less than 29% positive cells, ++= 30–59% positive cells, +++= 60–89% positive cells, or ++++= more than 90% positive cells. Low HIF1α expression was defined as “+” and “++,” while high HIF1α expression was defined as “+++” and “++++.” The staining intensity of HIF1α was performed by two experienced examiners who were blinded to the clinical data.

Estrogen receptor (ER), progesterone receptor (PR), Ki-67, HER2, and HIF1α were performed on paraffin-embedded BC tissues from biopsy. ER, PR, HER2, and Ki-67 were stained utilizing rabbit monoclonal antibodies SP1, EE2, 4B5 (F. Hoffmann-La Roche Ltd., Switzerland), and MIBI (Leica Biosystems Newcastle Ltd., United Kingdom). ER, PR, and Ki-67 staining were recorded as continuous variables. We defined positive nuclear staining more than 10% as ER- and PR-positive status. Positive HER2 status was indicated by a “+++” from the immunohistochemical evaluation result or positive result of fluorescence *in situ* hybridization (FISH) test for HER2. TNM staging was on the ground of the eighth American Joint Committee on Cancer criteria.

### Cell Culture and Reagents

MDA-MB-231 and MDA-MB-435 cell lines were obtained from the American Type Culture Collection (ATCC, Rockville, MD, United States) and were cultured in Dulbecco’s modified Eagle’s medium (DMEM) (Gibco) supplemented with 10% fetal bovine serum (FBS), and 1% penicillin/streptomycin (Life Technologies) under 37°C and 5% CO_2_ atmosphere incubator. Recombinant human IL-17A was purchased from Sigma. Paclitaxel was bought from MCE. ELISA (enzyme-linked immunosorbent assay) kit for human CXCL10 was obtained from R&D systems (Minneapolis, MN, United States).

### Hypoxia Condition

We incubated cells upon hypoxia (1% O_2_) condition in a glove box. After pre-exposure to low oxygen condition, we implemented all subsequent treatments in the glove box, preventing cellular damage from cell reoxygenation. Moreover, all procedures needed to change medium after hypoxic exposure; we pre-equilibrated the medium to the low-oxygen condition 24 h before use. Control cells just used normoxia-equilibrated medium.

### Hypoxia Inducible Factor-1α RNA Transfection

To decrease HIF1α expression, small interfering RNA (siRNA) was transfected to MDA-MB-231 and MDA-MB-435 cell with Lipofectamine RNAiMAX (Invitrogen). The sequences of the siRNA were listed as follows: siHIF1α-1, 5′-GGGCCACAAAGUCAGUAAAdTdT-3′; siHIF1α-2, 5′-GAGGAAGAACUAAAUCCAAdTdT-3′.

### Drug Sensitivity Assay

Breast cancer cells were seeded in a 96-well plate with 1 × 10^4^ cells/well. After adherence, the cells were treated with different concentrations of paclitaxel: 100, 50, 25, 12.5, 6.25, 3.13, 1.56, 0.78, 0.39, 0.20, 0.10, and 0 ng/ml). Cell activity was detected by CCK8 assay after 24 h. Fitted curves and 50% inhibition concentration (IC_50_) were used for comparing the sensitivity of the different groups.

### Quantitative Real-Time PCR

We extracted total RNA utilizing a TRIzol RNA extraction kit (Invitrogen, Carlsbad, CA, United States). The first-strand cDNA was synthesized using an PrimeScript RT Enzyme Mix (Takara RR036A). RT-qPCR was performed using a LightCycler LC480 instrument (Roche) according to the procedures of the manufacturer. 18S was used as internal control to normalize tests. Primer sequences are listed in Supplementary Table 1.

### Enzyme-Linked Immunosorbent Assay for Detecting CXCL10

We collected cell-conditioned medium and centrifuged it for 10 min. CXCL10 concentration in the supernatant was detected using an ELISA kit according to the protocol of the manufacturer.

### Bioinformatics Analysis

To analyze the potential immune-related pathway that HIF1α may take part in, we harvested HIF1α expression profiles for BC from The Cancer Genome Atlas (TCGA) database.^1^ Separating BC samples into high HIF1α expression and low HIF1α expression group with median expression of HIF1α, we screened differentially expressed genes (| FC| ≥ 2, FDR < 0.05) and performed The KEGG pathway analysis by the R (version 3.6.1) package “clusterProfiler.” We also analyzed GO annotation of the top 500 differentially expressed genes in terms of immune system process with Cytoscape (3.7.2) application “ClueGO.” To confirm the relationship between HIF1α and BC chemotherapy response, we analyzed GEO datasets GSE103787 and GSE50948. To avoid potential bias, we chose HR−/HER2+ BC patients given the same chemotherapy regimen. We accessed expression matrix and platform annotations with the R package ‘‘GEOquery.’’ The online tool Kaplan--Meier Plotter^2^ was utilized for exploring prognosis predicting the value of HIF1α.

### Statistical Methods

Categorical variables were analyzed by chi-square test, and continuous variables were analyzed by Student’s *t*-test. Continuous variables are depicted by mean ± SD (standard deviation). We analyzed the correlation between HIF1α expression and efficacy of NAC by univariate and multivariate logistic regression analysis. Nomogram was built based on multivariate logistic regression of continuous variables. The validity of the prediction model was examined by the area under the curve (AUC) of the receiver operating characteristic (ROC) curve and also the calibration curve. Nomogram was built with the R package “rms.” Factors included in the nomogram were screened by backward stepwise. ROC curve was performed by the R package “pROC.” The calibration curve of the prediction model was established by the R package “foreign.” R (version 3.6.1) and SPSS (version 23.0.0.0) were employed for statistical analysis. Two-sided *p* < 0.05 was ruled as statistically significant. Graphpad Prism 8, R (version 3.6.1), and Adobe Illustrator (version 21.0.0.0) were used to draw the photos in this work.

## Results

### Clinicopathologic Features of the Patients

Our current study totally included 108 patients, and their clinicopathologic characteristics are listed in Table 1. The mean age of the enrolled patients in the non-pCR group was 49.72 years old, while that in the pCR group was 49.09 years old. Representative immunohistochemistry staining of HIF1α expression is presented in Supplementary Figure 1. In the high HIF1α expression group, pCR rate was only 33.3% (21/63), while in the low HIF1α expression group, pCR rate achieved 60% (27/45) (Table 1, *p* < 0.001). Despite HIF1α staining, we also found that pCR rate after NAT was significantly correlated to ER status (*p* < 0.001), PR status (*p* = 0.008), Ki67 expression level (*p* < 0.001), HER2 amplification (*p* = 0.013), and histologic grade (*p* = 0.002) (Table 1). However, pCR rate had no significant relationship with T stage, clinical lymph node metastasis, and AJCC stage (Table 1).

**TABLE 1:** Baseline patient characteristics.

	**Level**	**Non-pCR**	**pCR (*n* = 32)**	***p***
		**(*n* = 76)**		
Age [mean (SD)]		49.72 (9.76)	49.09 (12.04)	^*^0.776
BMI [mean (SD)]		23.35 (2.72)	22.17 (2.54)	^*^0.044
ER [mean (SD)]		67.46 (33.28)	20.53 (33.72)	^*^<0.001
PR [mean (SD)]		49.34 (33.50)	30.91 (28.92)	^*^0.008
Ki67 [mean (SD)]		35.04 (21.74)	55.00 (19.18)	^*^<0.001
HER2 (%)	Negative	52 (68.4)	13 (40.6)	^**^0.013
	Positive	24 (31.6)	19 (59.4)	
Histologic grade (%)	0-II	45 (59.2)	8 (25.0)	^**^0.002
	III	31 (40.8)	24 (75.0)	
T stage (%)	I–II	17 (22.4)	11 (34.4)	^**^0.289
	III-IV	59 (77.6)	21 (65.6)	
Clinical lymph node metastasis	No	11 (14.5)	3 (9.4)	^**^0.684
	Yes	65 (85.5)	29 (90.6)	
AJCC stage	II	22 (28.9)	14 (43.8)	^**^0.205
	III	54 (71.1)	18 (56.2)	
HIF1α	High	42 (66.7)	21 (33.3)	^**^<0.001
	Low	18 (40)	27 (60)	

ER, estrogen receptor; PR, progesterone receptor; HER2, human epidermal growth factor receptor 2; pCR, pathological complete response, *Student’s *t*-test; **Pearson chi-square test.

### Logistic Regression Model of Pathological Complete Response and Nomogram to Predict Pathological Complete Response Rate for Those Who Received Neoadjuvant Therapy

**FIGURE 1:**
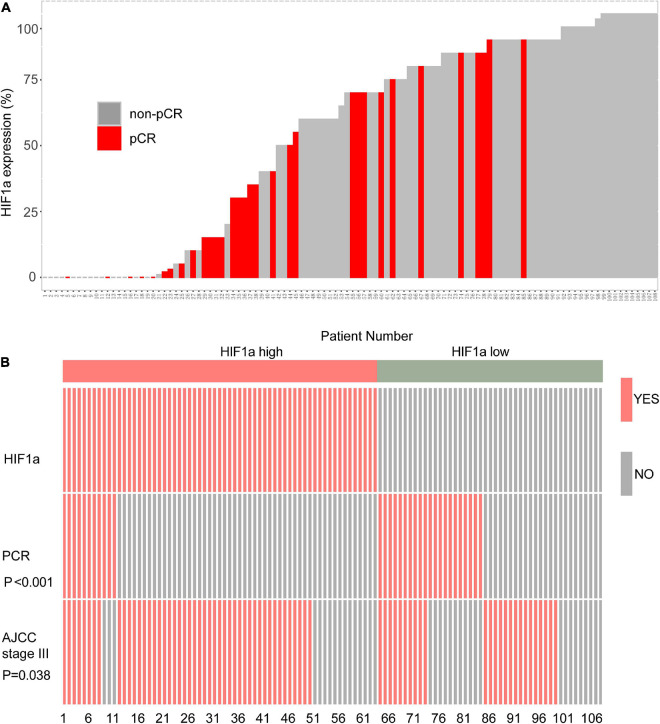
High hypoxia inducible factor-1α (HIF1α) staining is related with worse response of neoadjuvant therapy (NAT) containing paclitaxel and higher clinical stage in local advanced breast cancer (BC) cohort. **(A)** Bar plot showing NAT response and HIF1α staining results of each individual in Renji breast cancer NAT cohort. **(B)** Polymerase chain reaction (PCR) rate of NAT and ratio of patients reaching the AJCC stage III in different HIF1α expression groups. PCR, pathological complete response; ER, estrogen receptor; PR, progesterone receptor; HER2, human epidermal growth factor receptor 2; NAT, neoadjuvant therapy.

In the HIF1α low expression population, we can find that more individuals reached pCR. On the contrary, the individuals who reached pCR was relatively sparse in the high HIF1α expression population (Figure 1A). Then we performed analysis to further evaluate the correlation between the HIF1α expression and pCR rate and clinical stage. The results confirmed that pCR rate in the high HIF1α expression group was significantly lower than that in the low expression group (Figure 1B). Similarly, more patients reached stage III according to AJCC in the HIF1α high group than in the HIF1α low group (*p* = 0.038) (Figure 1B).

**FIGURE 2:**
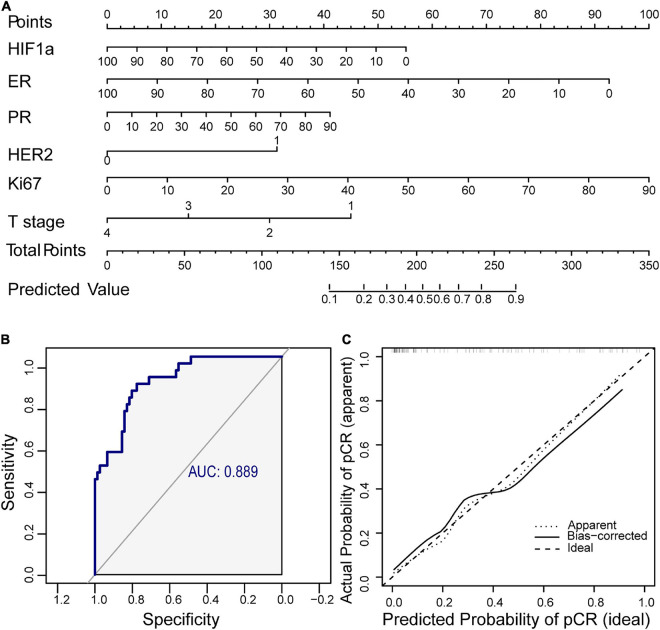
A nomogram including hypoxia inducible factor-1α (HIF1α) and clinicopathological factors is established for clinical prediction of NAT response. **(A)** Nomogram containing HIF1α and clinicopathological factors for predicting response of NAT containing paclitaxel. Factors included are chosen by backward stepwise. **(B)** The ROC of the nomogram. The AUC is calculated for evaluating the performance of the model. **(C)** Calibration curve of the prediction model. NAT, neoadjuvant therapy; ROC, receiver operating characteristic; AUC, area under the curve.

For clinical application of HIF1α in predicting NAT response, we established a nomogram combined with HIF1α and clinicopathological factors selected by backward stepwise selection and multivariate logistic regression analysis (Figure 2A). It showed that HIF1α expression level, ER expression level, Ki67 expression level, PR, HER2, and T stage were factors finally selected in the model. According to multivariate logistic regression analysis, low HIF1α expression was correlated with higher pCR rates (OR = 0.980, 95% CI 0.963–0.996, and *p* = 0.0155; Table 2). Low ER expression ratio were also correlated with higher pCR rates (OR = 0.967, 95% CI 0.944–0.986, and *p* = 0.0019; Table 2). What is more, high Ki67 level was correlated with higher pCR rates (OR = 1.041, 95% CI 1.013–1.075, and *p* = 0.0067; Table 2). Based on the above result, we first established ROC to confirm the predictive value of HIF1α expression in NAT response for BC patients: multivariate model combining HIF1α and clinicopathological features, which showed AUC 0.889, as shown in Figure 2B. To examine the discriminative ability of the model, we used bootstrap methods to calculate the mean absolute error with 1,000 repetitions. The mean absolute error was 0.04, and the calibration curve was also exhibited to evaluated goodness-of-fit of the model (Figure 2C), revealing good agreement between the predicted and actual probabilities of pCR.

**TABLE 2:** Predictive value for pCR of potential markers in breast cancer neoadjuvant chemotherapy cohort with logistic regression test.

**Variables**	**Univariate analyses**			**Multivariate analyses**		
	**OR**	**95% confidence interval**	***p***	**OR**	**95% confidence interval**	***p***
HIF1α	0.985	0.973 – 0.996	0.0076	0.980	0.963 – 0.996	0.0155
ER	0.967	0.954 – 0.979	<0.001	0.967	0.944 – 0.986	0.0019
PR	0.982	0.969 – 0.995	0.010	1.017	0.992 – 1.046	0.2120
HER2	3.167	1.361 – 7.600	0.008	3.142	0.989 – 10.86	0.0576
Ki67	1.043	1.022 – 1.066	<0.001	1.041	1.013 – 1.075	0.0067
T stage	0.548	0.327 – 0.891	0.018	0.578	0.295 – 1.08	0.0938

### Hypoxia Inducible Factor-1α Expression Can Predict Pathological Complete Response for Breast Cancer Patients After Receiving Neoadjuvant Therapy and Is Associated With Survival of Breast Cancer Patients

**FIGURE 3:**
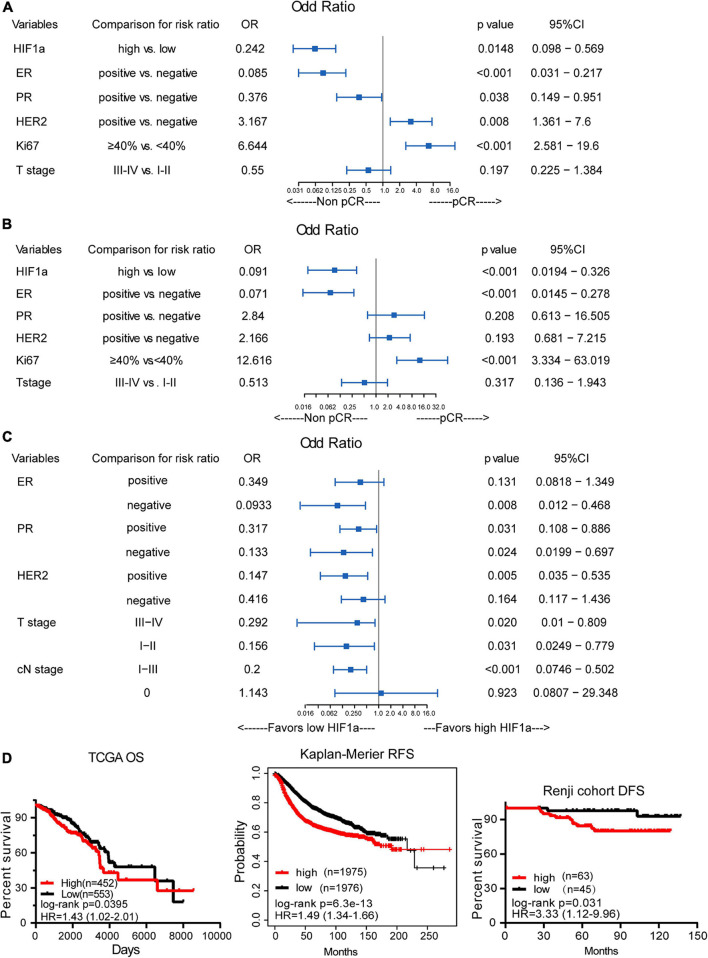
The correlation between hypoxia inducible factor-1α (HIF1α) expression and pathological complete response (pCR) and breast cancer survival. **(A)** Univariate logistic regression analysis of the relationship between HIF1α and pCR. **(B)** Multivariate logistic regression analysis of the relationship between HIF1α and pCR. **(C)** Subgroup analysis for the relationship of pCR and HIF1α expression levels using univariate logistic regression analysis. **(D)** High HIF1α expression predicts shorter overall survival (OS) for BC patients based on TCGA dataset (the left panel). High HIF1α expression predicts shorter relapse-free survival (RFS) for BC patients based on the analysis of the website of Kaplan–Meier (the middle panel). High HIF1α expression predicts shorter disease-free survival (DFS) for BC patients according to Renji cohort (the right panel); log-rank test.

Totally, 32 (29.6%) patients achieved a pCR after receiving NAT, among all the patients. In the non-pCR group, the mean HIF1α expression ratio was 60.91, while in the pCR group, the mean HIF1α expression ratio was 39.22, which indicated that a high HIF1α expression may predict lower pCR rate to NAT for BC patients (Table 1). HIF1α was also classified as categorized variable for further confirmation. Univariate logistic analysis (OR = 0.242, 95% CI 0.098–0.569, and *p* = 0.0148) and multivariate logistic analysis (OR = 0.091, 95% CI 0.0194–0.326, and *p* < 0.001) manifested that HIF1α was an independent predictive factor for the pCR rate to breast patients receiving NAT (Figures 3A,B). By univariate and multivariate logistic regression analysis, we found that ER status (OR = 0.071, 95% CI 0.0145–0.278, and *p* < 0.001) and Ki67 level (OR = 0.12.616, 95% CI 3.334–63.019, *p* < 0.001) were also a significantly predictive factor for pCR rate. These results suggested that patients with low HIF1α expression, negative ER status, and high Ki67 level may have higher pCR rate after NAT.

By subgroup analysis, we found that except in ER positive, HER2 negative, and cN0 stage subgroup, HIF1α expression all had the significance to predict pCR rate after NAT (Figure 3C). All the above consequences indicated that HIF1α expression had good significance to predict pCR rate after NAT for all BC patients.

Additionally, in HER2-positive patients, multivariate analysis showed that low HIF1α expression (OR = 0.048, 95% CI 0.0037–0.331, and *p* = 0.007), negative ER status (OR = 0.029, 95% CI 0.001–0.357, and *p* = 0.016), and high Ki67 expression level (OR = 12.181, 95% CI 1.933–131.116, and *p* = 0.017) were associated with higher pCR rate (Supplementary Figure 2).

Analysis for the correlation between HIF1α expression and OS based on the database from TCGA displayed that BC patients with high HIF1α expression had shorter OS (HR = 1.43, 95% CI = 1.02–2.01, and *p* = 0.0395), as shown in Figure 3D. Other analysis about RFS of BC patients who received neoadjuvant chemotherapy also revealed that high HIF1α expression was significantly associated with bad relapse-free survival (RFS) (HR = 1.49, 95% CI = 1.34–1.66, and *p* < 0.001), which is based on the website of Kaplan–Meier, as shown in Figure 3D. We drew similar results by analyzing our cohort that HIF1α expression was a significantly negative prognostic factor to RFS (HR = 3.33, 95% CI = 1.12–9.96, and *p* = 0.031), as shown in Figure 3D.

### Hypoxia Inducible Factor-1α Knockdown Can Increase the Chemosensitivity of Paclitaxel for Breast Cancer Cells

**FIGURE 4:**
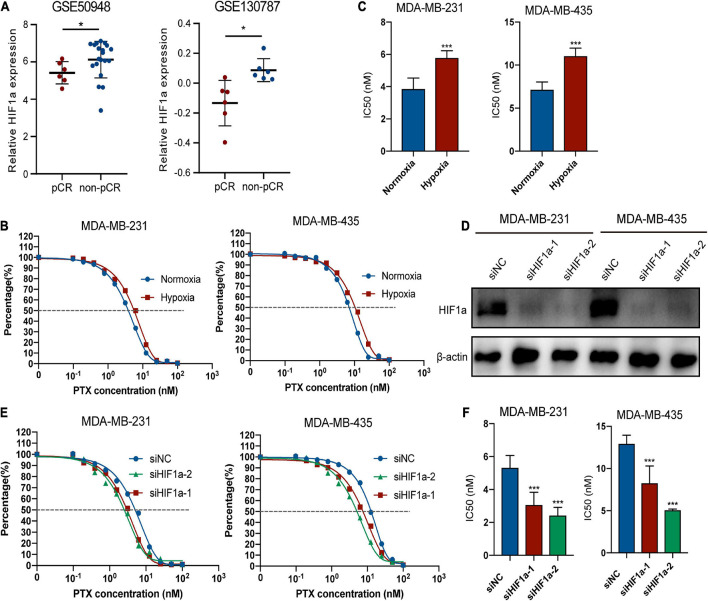
Hypoxia inducible factor-1α (HIF1α) can decrease the sensitivity of breast cancer cells to paclitaxel treatment. **(A)** Patients in the non-pCR group show higher HIF1α mRNA expression than those in the pCR group based on two GEO datasets about breast cancer neoadjuvant treatment containing usage of taxane in the research: GSE50948 (Expression Data From transNOAH Breast Cancer Trial) and GSE130787 (A Phase II Randomized Trial of Neoadjuvant Trastuzumab or Lapatinib or the Combination of Trastuzumab and Lapatinib, Followed by Docetaxel and Carboplatin with Trastuzumab and/or Lapatinib in Patients With HER2+ Breast Cancer). HIF1α expression was analyzed in the HR– HER2+ patients with the same treatment regimen and menstrual condition corresponding to the subgroup analysis and avoiding bias (Student’s *t*-test, * *p* < 0.05). **(B,C)** Effect of hypoxia in paclitaxel sensitivity of MDA-MB-231 and MDA-MB-435. Cells were treated with different concentrations of paclitaxel under normoxia or 24-h incubation of 1% hypoxia condition. CCK8 assay was applied for evaluating cell viability. **(B)** fitting curve of relative cell viability of two groups treated with different concentrations of paclitaxel. **(C)** IC_50_ of paclitaxel in different groups. Student’s *t*-test; values were defined as the mean ± SD. **(D)** Efficiency of HIF1α knockdown at the protein level. MDA-MB-231 and MDA-MB-435 cells transfected with small interference RNA (siRNAs)—siHIF1α-1, siHIF1α-2, or control (siNC) were pre-exposed to hypoxia for 24 h. Then we replaced the culture medium with fresh hypoxia conditioned medium, and cells were cultured for an additional 24 h under hypoxic condition. Finally, we collected the cells for Western blotting detection. **(E,F)** Influence of HIF1α inhibition on paclitaxel sensitivity of MDA-MB-231 and MDA-MB-435 under 1% hypoxia condition. MDA-MB-231 and MDA-MB-435 cells transfected with small interference RNA (siRNAs)—siHIF1α-1, siHIF1α-2, or control (siNC) were pre-exposed to hypoxia for 24 h. Then we replaced the culture medium with fresh hypoxia conditioned medium, and cells were treated with paclitaxel for an additional 24 h under hypoxic condition. CCK8 assay was applied for evaluating cell viability. **(E)** Fitting curve of relative cell viability of different groups treated with different concentrations of paclitaxel. **(F)** IC_50_ of paclitaxel in different groups. Student’s *t*-test; values were defined as the mean ± SD. *p < 0.05 and ***p < 0.001.

To better explore the relationship between HIF1α expression and chemosensitivity, we compared the PTX-pCR group with the PTX-non-pCR group BC patients from the GEO database (GSE50948 and GSE130787). To avoid bias, we analyzed patients of the same subtype (HR−/HER2+) based on the subgroup analysis before, who were also given the same chemotherapy regimen containing PTX. We found that patients in the non-pCR group had higher expression of HIF1α (Figure 4A). Because the analyzed patients were hormone receptor negative (HR−) and HER2-positive (HER2+) population, so we chose MDA-MB-231 and MDA-MB-435 cell lines for the following experiments. Figures 4B,C showed that hypoxia condition can decrease the sensitivity of MDA-MB-231 and MDA-MB-435 cells to PTX. To further examine the role of HIF1α on paclitaxel resistance, we knocked down HIF1α by transfecting siRNA upon hypoxia condition, and HIF1α interference efficiency is shown in Figure 4D. siHIF1α/MDA-MB-231 and siHIF1α/MDA-MB-435 cells were more sensitive to PTX with lower IC_50_ and cell survival rates than siNC cells (Figures 4E,F).

### Hypoxia Inducible Factor-1α Decreased PTX Chemosensitivity Potentially Through Regulating Interleukin 17 Signaling Pathway

**FIGURE 5:**
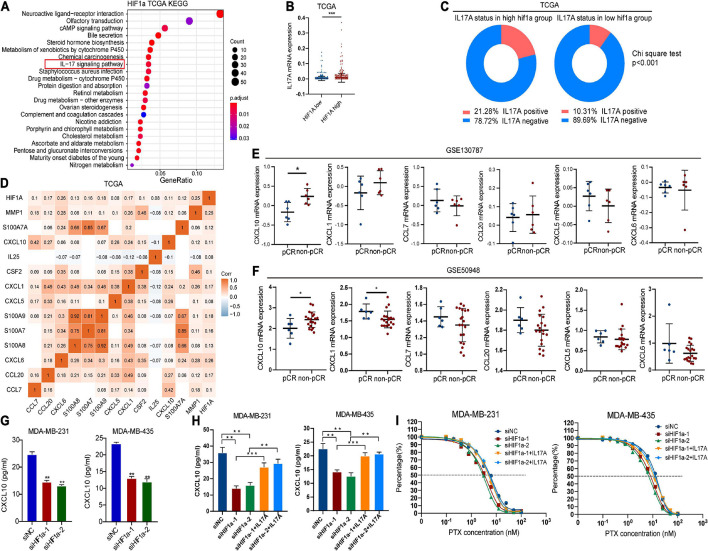
Hypoxia inducible factor-1α (HIF1α) influenced paclitaxel sensitivity through regulating the interleukin 17 (IL-17) signaling pathway. **(A)** KEGG pathway enrichment of differentially expressed genes in the HIF1A high group compared with the HIF1A low group. **(B)** IL-17A expression level in groups with different HIF1A expressions in TCGA breast cancer cohort. **(C)** Percentage of positive IL-17A mRNA expression (higher than 0) in groups with different HIF1A expressions in TCGA breast cancer cohort. **(D)** Correlation between HIF1A expression and DEGs participate in the IL-17 pathway in TCGA breast cancer dataset. White blocks without correlation coefficient means no statistically significant correlation (Pearson correlation) between two genes. **(E,F)** Expression of differentially expressed cytokines participating in the IL-17 pathway in groups with a different response group of paclitaxel-containing chemotherapy according to GEO datasets. **(G)** MDA-MB-231 and MDA-MB-435 cells transfected with small interference RNA (siRNAs)—siHIF1α-1, siHIF1α-2, or control (siNC) were exposed to hypoxia for 24 h. Then we detected the effect of HIF1α expression on CXCL10 expression using ELSIA assay. Student’s *t*-test, values were defined as the mean SD. **(H)** Effect of HIF1α suppression and human recombinant IL-17A on CXCL10 expression of breast cancer cells by ELISA assay. MDA-MB-231 and MDA-MB-435 cells transfected with siHIF1α-1, siHIF1α-2, or siNC were pre-exposed to hypoxia for 24 h. Then we replaced the culture medium with fresh hypoxia-conditioned medium, and cells were treated with paclitaxel and 100 ng/ml of recombinant human IL-17A for another 24 h. Then we collected the supernatant to detect the CXCL10 level. Student’s *t*-test; values were defined as the mean ± SD. **(I)** Fitted curve of paclitaxel in different groups. pCR, pathological complete response; IC_50_, 50% inhibition concentration. *p < 0.05; **p < 0.01; and ***p < 0.001.

To elucidate the underlying mechanism for high HIF1α expression contributing to chemotherapy resistance in BC, we performed KEGG analysis on the basis of TGGA data to analyze potential signaling pathways that HIF1α takes part in. Interestingly, we found that HIF1α was associated with IL-17 signaling pathway (Figure 5A), which indicates that HIF1α might possibly be relevant to immunoreaction in BC. The function of IL-17A is most well characterized among IL-17 family members (Xu and Cao, 2010). So we studied the association between HIF1α expression and IL17 expression from TCGA. We found that IL-17A expressed relatively higher in the HIF1α high group patients compared with that in the HIF1α low group patients (Figure 5B). Consistently, among the HIF1α high expression population, the IL-17A-positive population ratio was higher than that among the HIF1α low expression population (Figure 5C). In TCGA BC samples, HIF1α expression showed positive correlation with various cytokines and related genes participating in the IL17 pathway (Figure 5D). Furthermore, we also found that only the CXCL10 level in non-pCR patients was relatively higher than that in pCR patients both in GSE50948 and GSE130787 patient cohorts (Figures 5E,F). We demonstrated this result in *in vitro* experiments: both mRNA level and protein level of CXCL10 mRNA level increased under 1% hypoxia condition driving (Supplementary Figures 3A,B). CXCL10 expression level decreased as HIF1α interference (Figure 5G and Supplementary Figure 3C). We also conducted experiments to find the effect of IL-17A on CXCL10 expression. When HIF1α expression decreased, extraneous addition of IL-17A to BC cells can partly restore CXCL10 expression (Figure 5H). We also performed experiments to verify the role of IL-17A during the process of HIF1α resulting in PTX chemosensitivity. When adding recombinant human IL-17A into HIF1α knockdown BC cells, IL-17A could weaken the sensitivity of HIF1α interference to PTX for BC cells with higher IC_50_ (Figure 5I and Supplementary Figure 4). These findings partially prove that the role of HIF1α in PTX chemosensitivity might be through the HIF1α/IL17A/CXCL10 axis.

**FIGURE 6:**
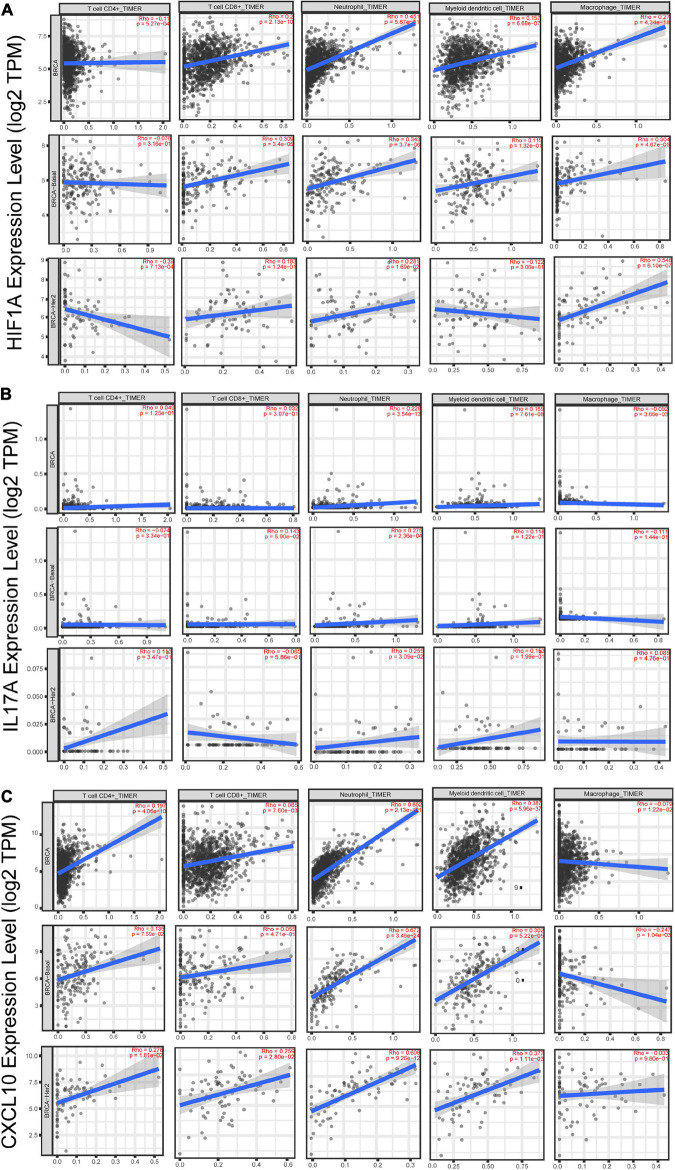
The correlation analysis between gene expression and infiltration of immune cells in breast cancer patients based on the website of timer (http://timer.cistrome.org/). **(A)** The correlation between HIF1α expression and CD4+ T cells, CD8+ T cells, neutrophils, dendritic cells, and macrophages in total BC, basal-like BC, and HER2-positive BC. **(B)** The correlation between IL-17A expression and CD4+ T cells, CD8+ T cells, neutrophils, dendritic cells, and macrophages in total BC, basal-like BC, and HER2-positive BCr. **(C**) The correlation between CXCL10 expression and CD4+ T cells, CD8+ T cells, neutrophils, dendritic cells, and macrophages in total BC, basal-like BC, and HER2-positive BC. The *X*-axis shows cell infiltration, and the *Y*-axis shows gene expression. Rho value and *p*-value were exhibited in red in each panel. Using the Spearman’s correlation analysis, we defined *R* > 0.25 and *p* < 0.05 as possessing significant correlation.

**FIGURE 7:**
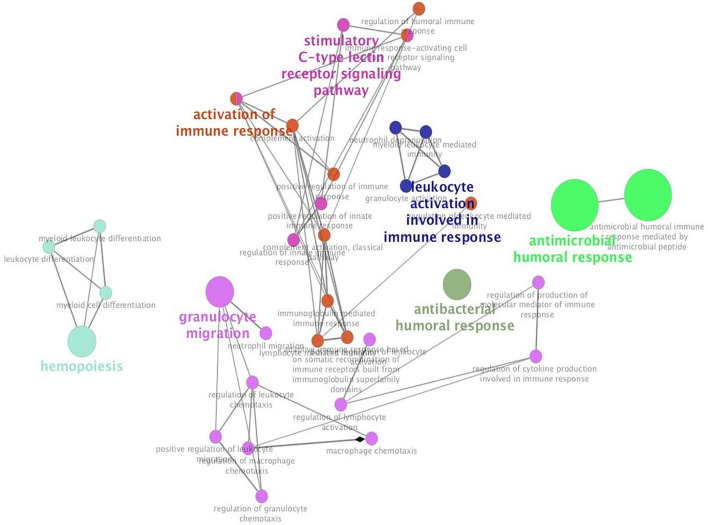
Hypoxia inducible factor-1α (HIF1α) can participate in leukocyte and granulocyte-related process. The top 500 differentially expressed genes screened from the HIF1α high group and low group from TCGA dataset are enriched with Cluego in Cytoscape in terms of GO immune system process annotation. Enriched pathways are clustered with different colors, and the size of enriched pathways is positively related with the number of enriched genes in different pathways.

Additionally, we studied the relationship between HIF1α, IL17A, and CXCL10 and immune microenvironment of BC. It is well known that TNBC and HER2+ BC are more tightly related to immune response (Shuai et al., 2020), so we primarily analyzed the association of these three genes with several immune cell subsets (CD4+ T cell, CD8+ T cell, neutrophil, dendritic cell, and macrophage) in TNBC and HER2+ BC population. Among these immune cells, only infiltration of neutrophils was positively correlated with HIF1α, IL17A, and CXCL10 expression, while other types of immune cells were weakly related to these three genes (Figures 6A–C). What is more, annotation of DEGs screened from HIF1α high and low groups was analyzed according to the GO Immune System Process. The results of the annotation showed that DEGs in HIF1α high samples were significantly enriched in the process related with granulocyte including regulating granulocyte migration, leukocyte chemotaxis, and cytokine production (Figure 7). It was reported that CXCL10 can recruit neutrophils to the disease site (Boztug et al., 2002). According to these results, we reasonably speculate that high HIF1α expression potentially contributes to PTX chemotherapy resistance by regulating IL17 pathway to attract more neutrophils to BC.

## Discussion

To better perform personalized treatment, researchers have dedicated to seek for effective predictive, and prognostic biomarkers in order to give individual patients a unique therapy. Our study first validated that high HIF1α expression was associated with low pCR rate and poor DFS in BC patients who received paclitaxel-based NAT. Next, we analyzed public database, which were also focused on paclitaxel-based chemotherapy and found that HIF1α expression was also a negative predictor of pCR after NAT for BC patients. In line with these findings, *in vitro* drug sensitivity assay validated that HIF1α could lead to paclitaxel chemo-insensitivity as well. Mechanistically, we discovered that HIF1α was involved in IL-17 signaling pathway *via* bioinformatic analysis based on public database and partially demonstrated this result through some *in vitro* experiments. All these results may provide us with novel insights into paclitaxel application in individual BC patients.

Our current work mainly drew three conclusions. First, we further reinforced the predictive value of HIF1α expression for BC patients after NAT. We testified the inverse relation of HIF1α expression with pCR rate in HER-positive and ER-negative subgroup patients for the first time. Second, we first used univariate and multivariate Cox analysis to demonstrate that overexpressed HIF1α can predict poor DFS. Third, we revealed that HIF1α potentially contributes to paclitaxel resistance by modulating IL-17 signaling pathway, and the HIF1α/IL17/CXCL10 axis may be related to neutrophil infiltration into HER2+ and HR− BC TME.

To our best knowledge, we initially performed subgroup analysis and found that high HIF1α expression can predict lower pCR rate in the ER negative group, PR negative or positive group, HER2 positive group, and lymph node metastatic group of patients. Through multivariate logistic model, we first revealed that HIF1α amplification can be an independent predictive biomarker for NAT in BC patients with HER2 positive and lymph node metastasis. It was reported that HIF1α overexpression was prone to exist in the HER2-positive population (Yehia et al., 2015; Badowska-Kozakiewicz et al., 2016) and lymph node metastatic patients (Gruber et al., 2004). Interestingly, HIF1α expression can promote CD73 activation (Hatfield and Sitkovsky, 2016), which leads to trastuzumab resistance in BC patients (Turcotte et al., 2017). In our study, HER2-positive patients were all given trastuzumab treatment; therefore, HIF1α may stimulate CD73 expression resulting in trastuzumab potency being lost and pCR rate compromised. These findings at least partly explain why high HIF1α expression can predict better pCR rate after NAT in HER2 positive/lymph node positive subgroup of patients.

In addition, our results also indicated that decreased expression of HIF1α can be a positive predictive factor for NAT response in ER-negative patients. HIF1α status and ER status may form a reciprocal loop mutually contributing to the aggressive feature for BC (Trastour et al., 2007). To confirm the association between HIF1α and ER status, we also performed KEGG analysis and found that HIF1α is involved in steroid hormone biosynthesis. Accordingly, these signaling pathways may clarify the reason that HIF1α can predict poor pCR rate in ER-negative patients to some extent, which needs further experiments to verify.

In this work, using Cox proportional hazard model, we attested that HIF1α expression can independently act as a prognostic biomarker for poor DFS for the first time for BC patients who received NAT. Although scarce studies focused on the relationship between HIF1α expression and NAT in BC patients, there were many studies about the impact of HIF1α positivity in BC regardless of the therapeutic methods. Oberhuber G et al. reported that HIF1α overexpression in lymph node-positive BC can be an independent predictive factor for DFS (Schindl et al., 2002); Increased HIF1α expression in lymph node-positive patients was related to shorter DFS (Gruber et al., 2004; Kronblad et al., 2006). Moreover, data from the Kaplan–Meier website showed that strong HIF1α expression was related to shorter RFS of patients receiving NAT, and this result was also demonstrated by an existing report (Nie et al., 2018). TCGA data demonstrated that high HIF1α expression in breast tumors was associated with poor prognosis (Chia et al., 2001). All these consequences indicated whatever the background of the BC was, HIF1α can be a prognostic biomarker, and concurrent administration of HIF1α inhibitor combined with traditional drugs can be an effective therapeutic schedule for patients with high HIF1α expression in tumors.

Hypoxia inducible factor-1α overexpression, a common feature for solid tumors, is tightly associated with BC growth (Semenza, 2010), vascularization (Forsythe et al., 1996), metastasis (Semenza, 2003; Zhang et al., 2012) as well as drug resistance (Jogi et al., 2019; Xu et al., 2019). It has been proven that an inhibitor targeted at HIF1 such as digoxin can block BC progression (Zhang et al., 2012) and enhance paclitaxel or gemcitabine chemosensitivity by regressing breast cancer stem cell (BCSC) enrichment (Samanta et al., 2014). Here we elucidated that HIF1α potentially regulates the immune condition of a tumor, especially the IL-17 pathway. ER− and HER2+ BC biopsies were infiltrated with more IL-17A-producing immune cells primarily comprising of lymphocytes and macrophages than ER+ BC biopsies (Chen et al., 2013; Cochaud et al., 2013). IL17A can activate the ERK1/2 pathway resulting resistance of BC cells to docetaxel treatment (Cochaud et al., 2013). The CXCL10 is one of the members belonging to the IL-17 signaling pathway. CXCL10 interacts with its receptor CXC motif chemokine receptor 3 (CXCR3) triggering a series of immune responses (Bonecchi et al., 1998). Upregulating CXCR3 and CXCL10 in MDA-MB-435 and MCF-7 cells can promote their invasive ability (Datta et al., 2006). Abrogating NF-κB/CXCR3 and CXCL10 axis in TNFα treatment MDA-MB-231 cells can inhibit their invasion capability (Choi et al., 2018). This study focused on a new mechanism that HIF1α overexpression induced IL17A expression decreasing MDA-MB-231 and MDA-MB-435 sensitivity to paclitaxel treatment.

Although our study makes some contributions to the role of HIF in taxane-based chemotherapy for BC, there still exist several limitations. First, due to the relative short follow-up time and a small number of enrolled patients, we failed to conclude the correlation between HIF1α expression and OS of patients with NAT. Second, there was a distribution bias in the subgroup of patients so that we cannot establish a multifactor model to predict the value of HIF1α in the ER± subgroup. Accordingly, we need to augment the enrolled population of breast patients and prolong the follow-up time to observe the prognostic role of HIF1α in OS among patients who received NAT. Furthermore, when selecting patients into the clinical trial, we should do our best to assure the balancing distribution of every subtype of BC. Third, we depicted that the neutrophils, which infiltrated, were the only kind of immune cells that correlated with HIF1α, IL17A, and CXCL10 expression through bioinformatic analysis. Profound investigation is needed to prove that the HIF1α/IL17/CXCL10 axis can recruit neutrophil infiltration to the BC TME leading to chemotherapy resistance.

Overall, our study confirmed the predictive and prognostic role of HIF1α expression in NAT for total BC patients. More than that, we revealed the essential role of the HIF1α/IL17 pathway in paclitaxel chemosensitivity.

## Data Availability Statement

The raw data supporting the conclusions of this article will be made available by the authors, without undue reservation.

## Ethics Statement

The studies involving human participants were reviewed and approved by the Institutional review board of Renji Hospital, School of Medicine, and Shanghai Jiao Tong University. The patients/participants provided their written informed consent to participate in this study.

## Author Contributions

JL, WY, and LY designed the study. HD and XS performed molecular biological experiments. HD and XS draftedthe manuscript and conducted the statistical analysis. LZ, YL, YD, FY, RS, and JP collected sample tissuesand clinical information. JL, LY, and YW revised the manuscript. All authors contributed to the article and approved the submitted version.

## Conflict of Interest

The authors declare that the research was conducted in the absence of any commercial or financial relationships that could be construed as a potential conflict of interest.

## Publisher’s Note

All claims expressed in this article are solely those of the authors and do not necessarily represent those of their affiliated organizations, or those of the publisher, the editors and the reviewers. Any product that may be evaluated in this article, or claim that may be made by its manufacturer, is not guaranteed or endorsed by the publisher.

## Footnotes

1
https://portal.gdc.cancer.gov

2
http://kmplot.com/analysis/index.php?p=background
