# Supplementary material for: HIF1α Regulates IL17 Signaling Pathway Influencing Sensitivity of Taxane-Based Chemotherapy for Breast Cancer
Source: Front Cell Dev Biol. 2021 Sep 14;9:729965. doi: 10.3389/fcell.2021.729965 (PMC8476907; doi:10.3389/fcell.2021.729965)
Supplement: Supplementary file 1 [file Data_Sheet_1.docx]

**Supplementary files**

**Supplementary figures**

**
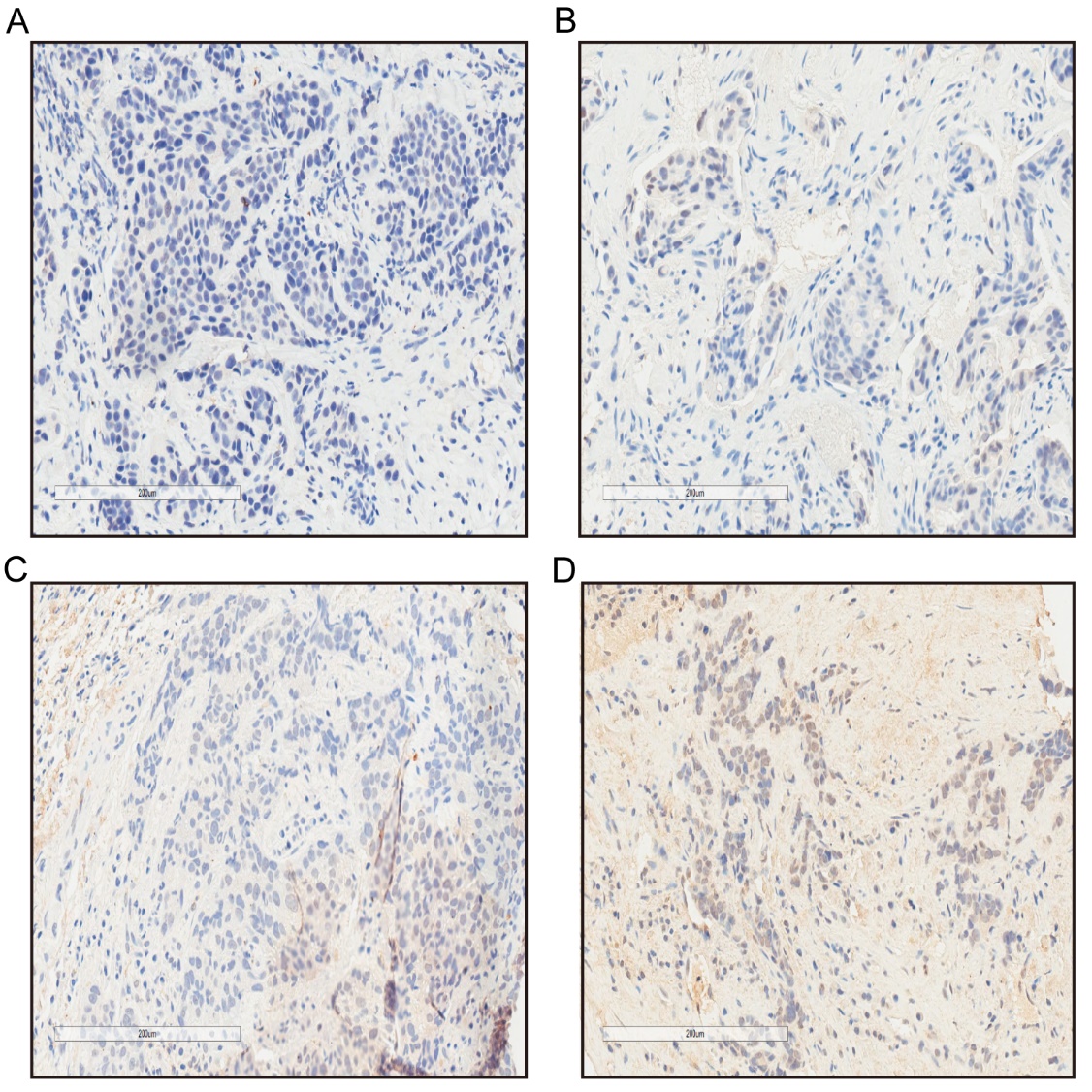
**

**Supplementary Figure 1**Representative image of immunohistochemistry staining of HIF1αexpressionin Breast cancer patients’ tissues

(A) HIF1αexpressionlevelrepresented as “+”; (B)HIF1αexpressionlevelrepresented as “++”; (C) HIF1αexpressionlevelrepresented as “+++”; (D) HIF1αexpressionlevelrepresentedas“++++”

**
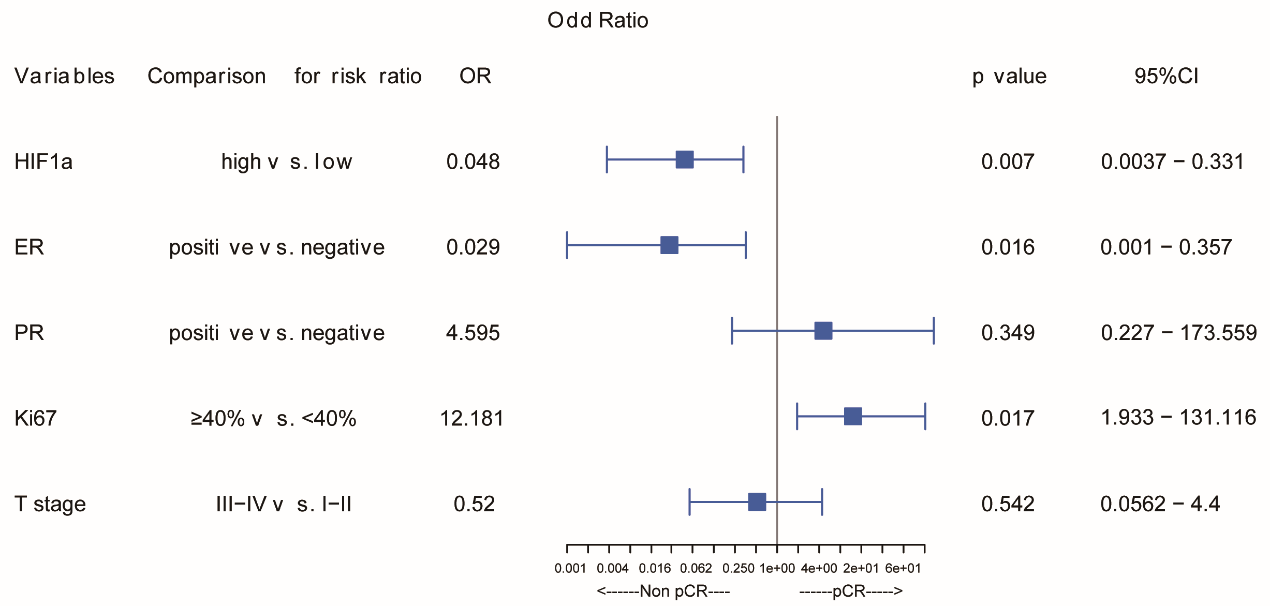
**

**Supplementary Figure 2** Analysis of correlation HIF1α expression and pCR for HER2 positive breast cancer patients

**
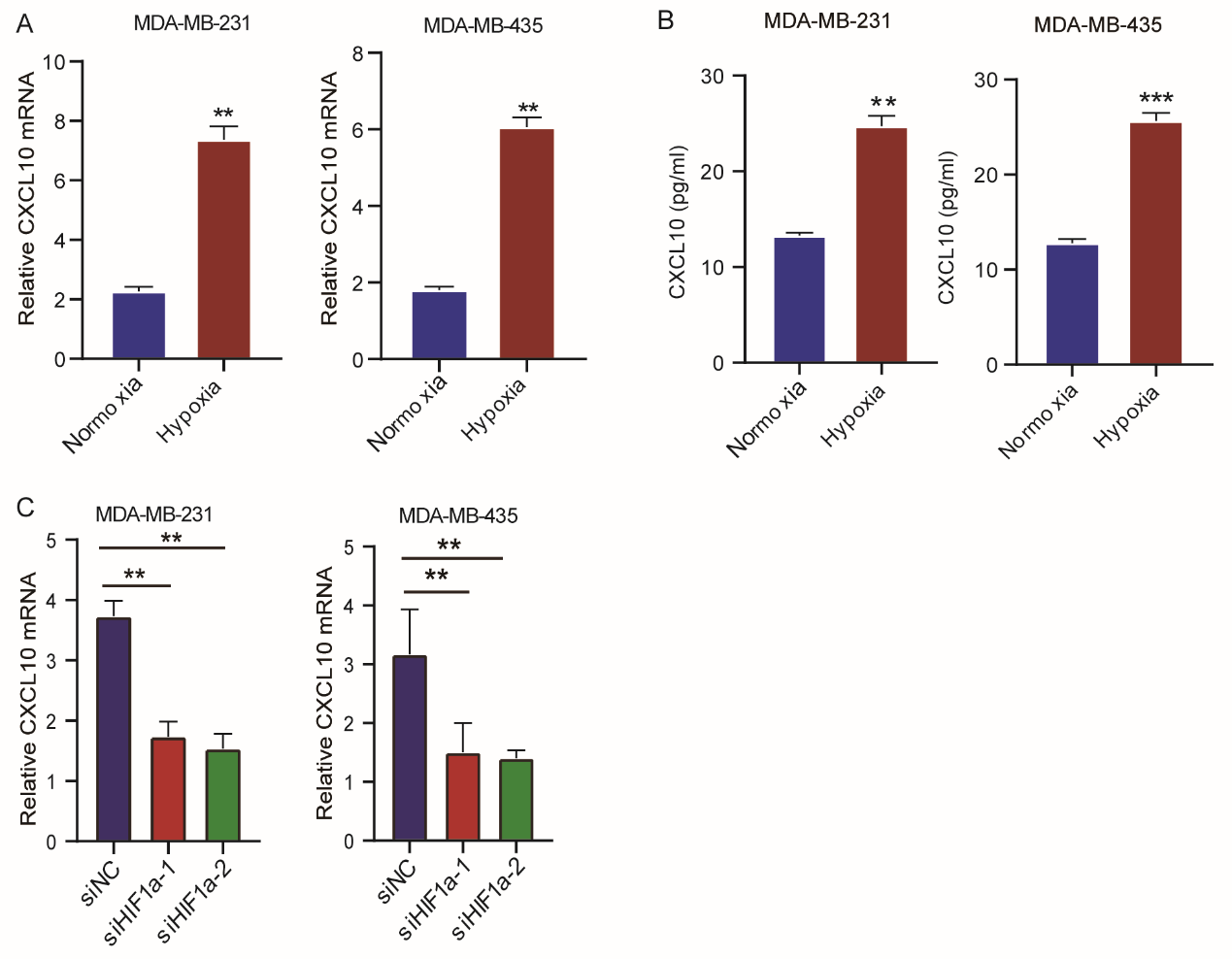
**

**Supplementary Figure 3**Relative expression of CXCL10 under hypoxia condition or HIF1a inhibition.

(A),(B) MDA-MB-231 and MDA-MB-435 cells were culturedunder normoxia or of 1% hypoxia condition for 4h. Then we detected CXCL10 mRNA and protein level by RT-pCR and ELISA assays.

(C)MDA-MB-231 and MDA-MB-435 cells transfected with small interference RNA (siRNAs) - siHIF1α-1, siHIF1α-2 or control (siNC) were exposed to hypoxia for 24 h. Then we detected the effect of HIF1α expression on CXCL10 expression using RT-PCR assay.

Student’s t test, values were defined as the mean ± SD.

** p<0.01; *** p<0.001.


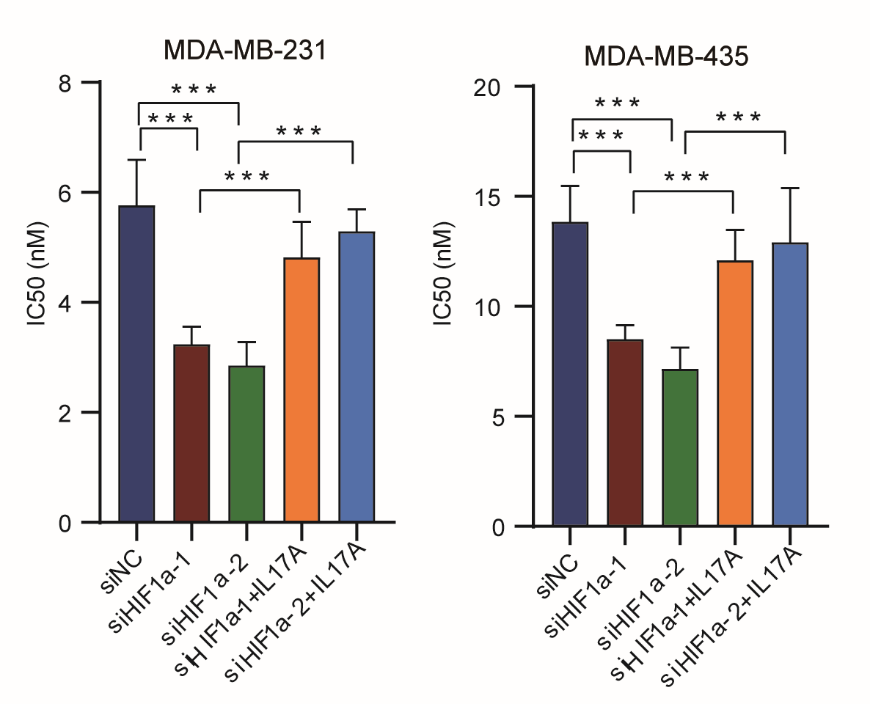


**Supplementary Figure 4** IC50 of paclitaxel in different groups. MDA-MB-231 and MDA-MB-435 cells transfected with siHIF1α-1, siHIF1α-2 or siNC were pre-exposed to hypoxia for 24 h. Then we replaced culture medium withfresh hypoxia conditioned medium, and cells were treated with paclitaxel and 100 ng/ml recombinant human IL-17A for another 24 h. Then we detected cells viability using CCK8 assay and drew fitted curve of paclitaxel in different groups. Student’s t test, values were defined as the mean ± SD.*** p<0.001.

| Gene name | Forward sequence (5’-3’) | Reverse sequence (5’-3’) |
| --- | --- | --- |
| 18S | TGCGAGTACTCAACACCAACA | GCATATCTTCGGCCCACA |
| CXCL10 | TGACTTCAGAACTGCGTATGCCATC | TCCCAAGATTGCCGTTTCCTAAAGAG |

**Supplementary Table 1** primer sequences of targeted genes.
